# Recognition of pseudoinvasion in colorectal adenoma using spatial glycomics

**DOI:** 10.3389/fmed.2023.1221553

**Published:** 2024-01-15

**Authors:** Fanny Boyaval, Arantza Fariña-Sarasqueta, Jurjen J. Boonstra, Bram Heijs, Hans Morreau

**Affiliations:** ^1^Department of Pathology, Leiden University Medical Center, Leiden, Netherlands; ^2^Center for Proteomics & Metabolomics, Leiden University Medical Center, Leiden, Netherlands; ^3^Department of Pathology, Amsterdam University Medical Centers, University of Amsterdam, Amsterdam, Netherlands; ^4^Department of Gastroenterology and Hepatology, Leiden University Medical Center, Leiden, Netherlands

**Keywords:** mass spectrometry imaging, colorectal cancer, pseudoinvasion, glycomic, marker

## Abstract

Pseudoinvasion (PI) is a benign lesion in which cancer is mimicked in the colon by misplacement of dysplastic glands in the submucosa. Although there are morphological clues, the discrimination of PI from true invasion can be a challenge during pathological evaluation of colon adenomas. Both overdiagnosis and underdiagnosis can result in inadequate clinical decisions. This calls for novel tools to aid in cases where conventional methods do not suffice. We performed mass spectrometry imaging (MSI)-based spatial glycomics analysis on a cohort of formalin-fixed paraffin-embedded tissue (FFPE) material from 16 patients who underwent polypectomy. We used this spatial glycomic data to reconstruct the molecular histology of the tissue section using spatial segmentation based on uniform manifold approximation and projection for dimension reduction (UMAP). We first showed that the spatial glycomic phenotypes of the different morphological entities separated as distinct clusters in colon tissues, we separated true invasion from the other morphological entities. Then, we found that the glycomic phenotype in areas with suspected PI in the submucosa was strongly correlating with the corresponding glycomic phenotype of the adenomatous colon epithelium from the same tissue section (Pearson correlation distance average = 0.18). These findings suggest that using spatial glycomics, we can distinguish PI as having a molecular phenotype similar to the corresponding surface epithelium and true invasion as having a different phenotype even when compared to high-grade dysplasia. Therefore, when a novel molecular phenotype is found in the deepest submucosal region, this may be used as an argument in favor of true invasion.

## 1 Introduction

Discrimination of so-called pseudoinvasion (PI) from true invasion is an outstanding diagnostic challenge during pathological evaluation of resected colon polyps. PI is a benign lesion in which cancer is mimicked by misplacement of dysplastic epithelium in the submucosa. PI is diagnosed pathologically after endoscopic polypectomy of colon adenomas, especially in pedunculated polyps. However, in difficult cases, the inadequate recognition of PI can lead to overdiagnosis and consequently to undesirable colonic surgical resections which can affect the quality of life of the patients (1). Since the introduction of population screening, early stage colorectal lesions are far more often diagnosed than ever before (Detection at early stage 67%). To date, there is a lack of reliable statistics results that highlight the frequency of PI occurrence, however it is likely that every GI pathologist is confronted with this critical issue. Even though there are differentiating histopathological criteria available, such as the absence of a desmoplastic reaction and the presence of iron (a sign of early bleeding) in the case of PI these are not always present or evaluable. Hence, expert panels can be needed to provide trustworthy and accurate assessments (2). This calls for novel, specific and sensitive tools to aid in cases where conventional methods do not suffice.

N-glycans, a common form of post-translational protein modification, are known to play a significant role in fundamental molecular and cellular processes involved in cancer such as tumor cell invasion, cell motility, formation of metastases, angiogenesis, and modulation of the immune response (3, 4). In colorectal cancer, the alterations observed in the *N*-glycome have also demonstrated the ability to differentiate between distinct morphological subtypes of early-stage, while also displaying tumor stage specificity (5, 6). We have previously reported the use of mass spectrometry imaging (MSI)-based spatial glycomics analysis of formalin-fixed paraffin-embedded tissue (FFPE) to differentiate morphological entities in early colorectal cancer (7, 8). Here, we have applied the same strategy to demonstrate that using spatial glycomic profiling we were able to show the similarity of the glycomic phenotype in areas suspected to have PI in the submucosa and the corresponding glycomic phenotype of the adjacent colon epithelium.

## 2 Materials and methods

### 2.1 Tissue sample collection

Endoscopic polypectomy specimens (*n* = 16) used in this study were obtained from the Department of Pathology of the Leiden University Medical Center. Tissues were formalin-fixed and paraffin-embedded (FFPE) following routine diagnostic protocols. All samples were anonymized, according to the national ethical guidelines (“Code for Proper Secondary Use of Human Tissue,” Dutch Federation of Medical Scientific Societies).

### 2.2 Sample preparation for spatial N-Glycomics by MALDI-MSI

FFPE tissues were sectioned at 6 μm thickness. Deparaffinization, rehydration and sialic acid linkage-specific derivatization were performed as described previously (8). Tissue enzymatic *N*-glycan release was performed applying 10 layers of PNGase F (0.1 μg/μL in Tris buffer) and CHCA matrix was used (5 mg/mL CHCA in 50:49.9/0.1 (% v/v/v) ACN:mQ:TFA).

### 2.3 Spatial glycomics and histopathological analysis

All specimens were mixed and measured in randomized fashion. Spatial glycomics was performed on a rapifleX MALDI-TOF/TOF-MS instrument in positive ion reflectron mode (Bruker Daltonics). Spectra were recorded using 1,000 laser shots per pixel with 50 × 50 μm^2^ pixel size (laser setting “M5 small”) over an *m/z* range of 900–3,300. After the analysis, excess MALDI-matrix was removed, tissues were hematoxylin and eosin (H&E) stained following routine histopathological procedures and then scanned using a digital slide scanner (IntelliSite Pathology Ultra-Fast Scanner, Philips, Eindhoven, Netherlands). In the flexImaging software, image scans and spatial glycomics data were co-registered and regions of interest (ROIs) were selected based on the histopathological analysis of the H&E slides by expert pathologists (AFS, HM). The spatial glycomics data, co-registered H&E images and ROIs from the whole cohort were imported into SCiLS lab software (2016b, version 4.01.8781, Bruker Daltonics).

### 2.4 Data preprocessing and statistical analysis UMAP

Data preprocessing were performed using the same methods as in Boyaval et al. (8). Spatial segmentation using uniform manifold approximation and projection for dimension reduction (UMAP) analysis was performed on peak picked, TIC normalized single-pixel spectra, exported from SCiLS Lab for each individual tissue. UMAP was performed using the *Seurat v3* package in R using default parameters. Each pixel were translated to RGB colors corresponding the color of the UMAP cluster, cluster corresponding to non-cellular areas were colored as white in the main figure. Molecular histology images were reconstructed in MATLAB. The correlation-based distance measures were performed using Pearson methods in R for each single tissue between the different morphologies by using the average intensity of the ROIs. In the Pearson correlation distance, similar object close to one other have a score close to 0, the bigger the distance the higher the score.

## 3 Results and discussion

We performed spatial glycomics on FFPE material of 16 patients who underwent a polypectomy and reconstructed the molecular histology of the tissues using spatial segmentation based on uniform manifold approximation and projection for dimension reduction (UMAP). Molecular histology reconstruct-images were compared with the corresponding post-MSI H&E staining to evaluate the molecular signatures of areas with suspected PI.

### 3.1 Differentiation between invasive vs. non-invasive adenocarcinoma

**Figure 1:**
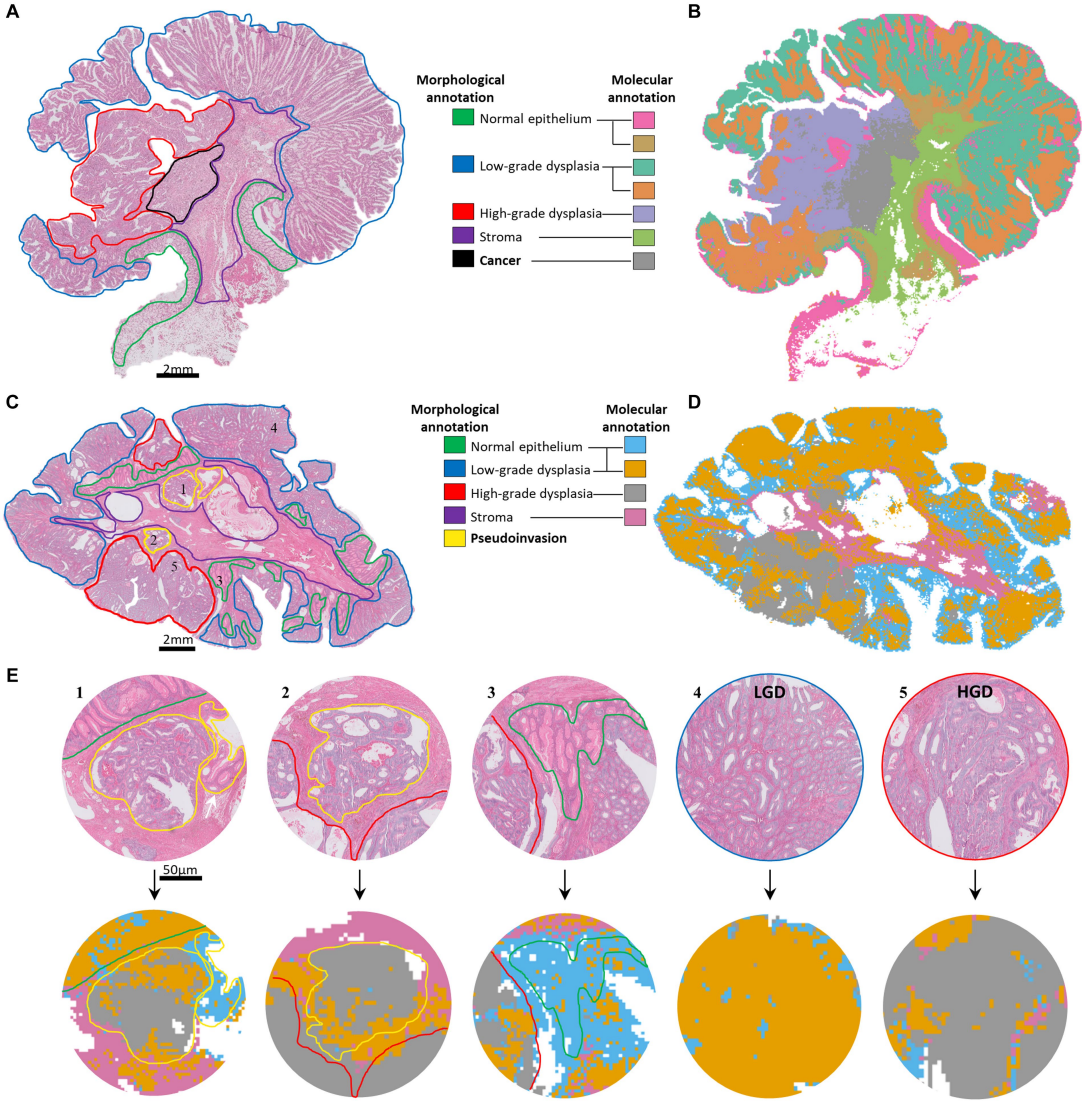
**(A)** Tissue-section 5: H&E staining of a T1 colorectal cancer with invasive adenocarcinoma with the histological annotations on the right. **(B)** Molecular histology reconstruct-image of the same tissue after UMAP analysis on the spatial glycomics data with the corresponding annotations on the left. **(C)** Tissue 9: H&E staining and **(D)** molecular histology reconstruct-image of a tissue with confirmed PI and histological annotation in the middle. **(E)** Zoom in (40x) on five areas in tissue **C**, with corresponding numbers to the areas in **C**. Example in E1, green line in H&E; normal epithelium appeared as orange/blue in the molecular histology reconstruct-image. Yellow line in H&E; pseudoinvasion appeared as grey (same as high-grade dysplasia) and as blue (same as normal epithelium) in the molecular histology reconstruct-image.

To test that spatial glycomics was capable of differentiating invasive adenocarcinoma from (non-invasive) dysplastic areas in colorectal polyps, eight cases of T1 colorectal cancer (malignant polyp) were analyzed (Table 1). In seven cases, the histological annotations of the invasive areas directly showed the expected molecular profiles. In Figure 1A, a representative case with invasive adenocarcinoma, high-grade, low-grade dysplasia and normal epithelium is depicted (Tissue-section 5, Table 1). Each of these morphological entities corresponded to a specific molecular signature as shown in the molecular histology reconstruct-image in Figure 1B.

**Table 1:** Clinical characteristics of patient cohort for tissue with true invasion in colorectal adenocarcinoma.

Tissue true invasion	1	2	3	4	5	6	7	8
Cancer: histological annotation	Yes	Yes	Yes	Yes	Yes	Yes	Yes	Yes
Cancer: molecular annotation	Yes	Yes	Yes	Mucinous pool	Yes	Yes	Yes	Yes
Gender	Female	Male	Male	Male	Female	Male	Male	Male
Age (years)	59	65	57	67	69	71	67	71
Tumor differentiation	Well/moderately	Well/moderately	Well/moderately	Well/moderately	Well/moderately	Well/moderately	Well/moderately	Well/moderately
Lymphovascular invasion	No	Yes	Yes	No	Yes	Yes	No	Suspicious
Tumor budding	No	Grade 3	Grade 1	No	Grade 2	Grade 2	No	Grade 1
Kikuchi/ Haggit level	sm3	sm1	sm2	n/a	Haggit 2–3	sm2	sm1	sm2
LNM (1-yes/0-no)	1	1	1	0	0	0	0	1
Diameter (cm)	1.5	0.9	1.2	1	0.6	0.9	0.7	0.6
Depth (mm)	5	2	2	3	6	5	2	2
Endoscopy morphology	Sessile	Sessile	Sessile	Sessile	Pedunculated	Sessile	Sessile	Sessile
Topography	Rectosigmoid	Rectosigmoid	Rectum	Transversal	Sigmoid	Sigmoid	Rectum	Sigmoid
TNM (+/− resection)	pT0N1	pT0N1(mi)M0	pT0N1	pT4N1	pT1N0	pT1 (NxMx)	pT1 (NxMx)	pT1 (NxMx)

In one case (Tissue 4, Supplementary Figures S1A,B and Table 1), the molecular signature of the invasive part did not fully match the histological annotation but highlighted the presence of mucinous pools with invasive cells as a novel separate cluster in the submucosa.

### 3.2 Molecular-histology features of pseudoinvasion

Then a total of eight cases with suspected PI were analyzed (Table 2). In seven cases, the morphological appearance of the suspected PI areas was similar to the one of the surface low-grade dysplasia epithelium (Supplementary Figures S1C,D). Rather than showing up as a distinct molecular phenotype, as would be expected for true invasive adenocarcinoma, the molecular histology of the suspected PI areas correlated highly with the histologically confirmed low-grade dysplasia areas in the same tissue (Pearson correlation distance = 0.05; Table 2 and Supplementary Table S1).

**Table 2:** Clinical characteristics of patient cohort for tissue with pseudoinvasion, *N* = Normal epithelium; LG = low-grade dysplasia; HG = High-grade dysplasia.

Tissue Pseudoinvasion	9	10	11	12	13	14	15	16
Pseudoinvasion: histological annotation	HG & N	LG	LG	LG	LG	LG	LG	LG
Pseudoinvasion: molecular annotation	HG & N	LG	LG	LG	LG	LG	LG	LG
Pearson correlation Distance	0.01 & 0.06	0.09	0.23	0.05	0.14	0.03	0.20	0.22
Gender	Male	Male	Male	Male	Male	Male	Male	Male
Age	75	62	71	60	81	60	64	62
Diameter (cm)	2.4		1.2	1.5	1.7	1.7	1.8	2
Endoscopy morphology	Pedunculated	Sessile	Pedunculated	Pedunculated	Sessile	Pedunculated	Pedunculated	Pedunculated
Topography	Sigmoid	Sigmoid	Sigmoid	Sigmoid	Descending	Sigmoid	Sigmoid	Sigmoid

In another case, in Figures 1C,E (Tissue 9, Table 2), PI of normal and high grade dysplastic epithelium correlated thoroughly with the corresponding molecular histology in Figures 1D,E (Pearson correlation distance = 0.01 & 0.06).

### 3.3 Discussion

Here, the application of MALDI-MSI-based spatial glycomics has been used to address the differential diagnosis of PI from true invasive cancer.

Dimensionality reduction on the high-dimensional spatial glycomics data provided molecular histology. This molecular histology showed comparability to conventional histomorphology in recognizing normal and different grades of neoplastic epithelium, but it displayed enhanced discriminatory potential between PI and true invasion.

In this report we showed that in all analyzed cases the glycomic molecular phenotype of PI was identical to that of the corresponding surface epithelium (either high-grade dysplasia, low-grade dysplasia or normal epithelium). We also highlight that the same technology is able to detect true invasion as separate molecular phenotype even with a high correlation score with the high-grade dysplasia (Pearson correlation distance = 0.26; Supplementary Table S1). Therefore, when a novel molecular phenotype is found in the deepest submucosal part, it might be used as an argument in favor of true invasion. These findings are in line with our previous studies that showed strong changes in glycomic signatures during the progression of stage I and stage II colorectal cancer (8). Additionally, others have demonstrated that employing the same technique has the potential to enhance tumor classification accuracy in pancreatic, ovarian, and hepatocellular carcinoma (9–11).

The fact that FFPE material is suitable for the spatial glycomics platform, and that the total diagnostic time spent is comparable to most immunohistochemistry protocols (approximately 2 days), makes this technique appropriate for running in parallel with the routine diagnostic workflow. The molecular histology reconstruct-image resulting from the spatial glycomics platform facilitates the direct comparison with H&E and other histopathological stainings by trained pathologists. However, while the samples, sample preparation, and required reagents for these analyses are easily obtainable and manageable within most pathology departments, it’s important to note that an imaging mass spectrometer and the expertise to operate the instrument and extract the data are necessary. The initial investment in the instrument and the requirement for specialized skills during operation should be carefully considered. For the data analysis part, once the pipeline and code are developed, it becomes user-friendly and applicable on any computer. This underscores the dual aspects of both accessibility and the requisite expertise associated with deploying this spatial glycomics platform in routine diagnostics. Nevertheless, this technique holds the potential to serve as a diagnostic tool in challenging scenarios where distinguishing between T1 colon cancer and PI is a subject of debate.

Clinical pathologists often pay more attention to morphological changes instead of the possible explanations behind them. Recently, an article proposed the concept of ecological pathology, stating that it is essential to apply the ecological (−evolutionary) principles and approaches to study the etiology, pathogenesis, pathological changes and outcomes of human diseases (12). For example, in this paper, tumor vascular mimicry is proposed to be an ecological adaptation. Similarly, the now discussed pseudoinvasion is a benign condition in which cancer is mixed in the colon by misplacement of dynamic lands in the submucosa, which might also be thought as an ecological adaptation due to the similar habitat.

This proof-of-concept study, while limited in cases, emphasizes the necessity for a more extensive cohort, particularly incorporating additional adenomas with high-grade dysplasia in the pseudoinvasion component, to validate these findings. Increasing the sample size holds the potential to significantly enhance statistical robustness, pattern generalizability, stability, and detection reliability. Such a comprehensive dataset is crucial for establishing a strong foundation, ensuring precision across cases, and minimizing the impact of variability in practically applying glycomic patterns to clinical diagnoses. While ongoing optimization of MSI analysis techniques remains valuable, and several studies have been working on it (13–16), the true strength of the separation criteria lies in the extensive representation of diverse cases through a larger sample size.

However, the presented results highlight the potential of spatial glycomics-based molecular histology as novel tool for routine diagnostics. We hope that this technical report will be a first steppingstone in this area with MSI based technology combined with dimensionality reduction algorithm.

## Data availability statement

The raw data supporting the conclusions of this article will be made available by the authors, without undue reservation.

## Ethics statement

The studies involving humans were approved by Leiden University Medical Center ethical committee. The studies were conducted in accordance with the local legislation and institutional requirements. The human samples used in this study were acquired from a by-product of routine care or industry. Written informed consent for participation was not required from the participants or the participants’ legal guardians/next of kin in accordance with the national legislation and institutional requirements.

## Author contributions

FB: conceptualization, data curation, formal analysis, investigation, methodology, software, validation, visualization, writing – original draft, and writing – review & editing. AF-S: conceptualization, data curation, resource, project administration, supervision, and writing – review & editing. JB: conceptualization, resource, supervision, writing – original draft, and writing – review & editing. BH: conceptualization, data curation, investigation, methodology, project administration, resources, supervision, validation, visualization, and writing – review & editing. HM: conceptualization, data curation, funding acquisition, methodology, project administration, resources, supervision, validation, and writing – review & editing. All authors contributed to the article and approved the submitted version.
