# Supplementary material for: Recognition of pseudoinvasion in colorectal adenoma using spatial glycomics
Source: Front Med (Lausanne). 2024 Jan 15;10:1221553. doi: 10.3389/fmed.2023.1221553 (PMC10822882; doi:10.3389/fmed.2023.1221553)
Supplement: Supplementary file 1 [file Data_Sheet_1.docx]

Supplementary Material

Recognition of pseudoinvasion in colorectal adenoma using spatial glycomics

Fanny Boyaval^1,2^, Arantza Fariña-Sarasqueta^3^, Jurjen J. Boonstra^4^, Bram Heijs^2^, Hans Morreau^1^*

* Correspondence: Prof. Dr. Hans Morreau, [J.Morreau@lumc.nl](mailto:J.Morreau@lumc.nl)


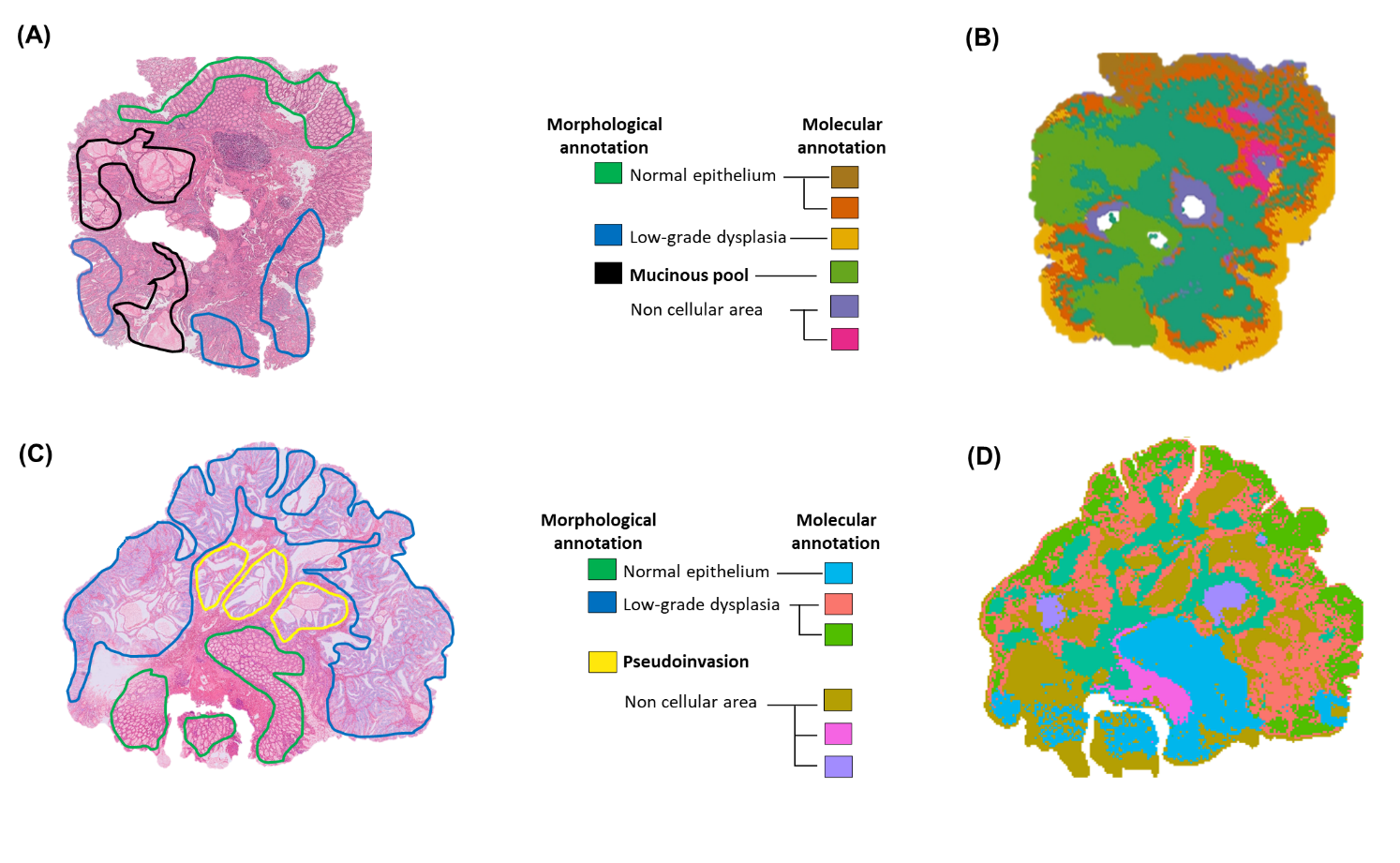


**Supplementary figure 1**: (**A**) Tissue 10: H&E slide of a T1 CRC with invasive adenocarcinoma and mucinous pool with the histological annotations on the right. (**B**) Molecular histology image of the same tissue after UMAP analysis on the spatial glycomics data with the corresponding annotations on the left. (**C**) Tissue 12: H&E slide of a PI tissue with the histological annotations on the right. (**D**) Molecular histology image of the same tissue with the corresponding annotations.

| **Tissue 5** |  | **HGD** | **CA** | **LGD** | **NE** |
| --- | --- | --- | --- | --- | --- |
|  | **HGD** | 0.00 | 0.26 | 0.12 | 0.27 |
|  | **CA** | 0.26 | 0.00 | 0.19 | 0.11 |
|  | **LGD** | 0.12 | 0.19 | 0.00 | 0.09 |
|  | **NE** | 0.27 | 0.11 | 0.09 | 0.00 |
|  |  |  |  |  |  |
| **Tissue 9** |  | **NE** | **PI-NE** | **LGD** | **HGD** |
|  | **NE** | 0.00 | 0.06 | 0.30 | 0.53 |
|  | **PI-NE** | 0.06 | 0.00 | 0.35 | 0.43 |
|  | **LGD** | 0.30 | 0.35 | 0.00 | 0.32 |
|  | **HGD** | 0.53 | 0.43 | 0.32 | 0.00 |
|  | **PI-HGD** | 0.56 | 0.44 | 0.33 | 0.01 |
|  |  |  |  |  |  |
| **Tissue 10** |  | **NE** | **LGD** | **PI** |  |
|  | **NE** | 0.00 | 0.09 | 0.17 |  |
|  | **LGD** | 0.09 | 0.00 | 0.09 |  |
|  | **PI** | 0.17 | 0.09 | 0.00 |  |
|  |  |  |  |  |  |
| **Tissue 11** |  | **LGD** | **NE** | **PI** |  |
|  | **LGD** | 0.00 | 0.14 | 0.23 |  |
|  | **NE** | 0.14 | 0.00 | 0.05 |  |
|  | **PI** | 0.23 | 0.05 | 0.00 |  |
|  |  |  |  |  |  |
| **Tissue 12** |  | **NE** | **LGD** | **PI** |  |
|  | **NE** | 0.00 | 0.39 | 0.33 |  |
|  | **LGD** | 0.39 | 0.00 | 0.05 |  |
|  | **PI** | 0.33 | 0.05 | 0.00 |  |
|  |  |  |  |  |  |
| **Tissue 13** |  | **NE** | **LGD** | **PI** |  |
|  | **NE** | 0.00 | 0.37 | 0.47 |  |
|  | **LGD** | 0.37 | 0.00 | 0.14 |  |
|  | **PI** | 0.47 | 0.14 | 0.00 |  |
|  |  |  |  |  |  |
| **Tissue 14** |  | **NE** | **LGD** | **PI** |  |
|  | **NE** | 0.00 | 0.09 | 0.08 |  |
|  | **LGD** | 0.09 | 0.00 | 0.03 |  |
|  | **PI** | 0.08 | 0.03 | 0.00 |  |
|  |  |  |  |  |  |
| **Tissue 15** |  | **LGD** | **NE** | **PI** |  |
|  | **LGD** | 0.00 | 0.16 | 0.20 |  |
|  | **NE** | 0.16 | 0.00 | 0.13 |  |
|  | **PI** | 0.20 | 0.13 | 0.00 |  |
|  |  |  |  |  |  |
| **Tissue 16** |  | **PI** | **LGD** | **NE** |  |
|  | **PI** | 0.00 | 0.22 | 0.28 |  |
|  | **LGD** | 0.22 | 0.00 | 0.07 |  |
|  | **NE** | 0.28 | 0.07 | 0.00 |  |

Supplementary table: Tables of the Pearson correlation distance between morphologies (ROIs) in individual tissue. Invasive adenocarcinoma (CA), high-grade (HGD), low-grade (LGD) dysplasia, normal epithelium (NE), Pseudoinvasion (PI). Similar object close to one other have a score close to 0, the bigger the distance the higher the score
